# Neonatal Kidney Function, Injury and Drug Dosing: A Contemporary Review

**DOI:** 10.3390/children12030339

**Published:** 2025-03-07

**Authors:** Eveline Staub, Srinivas Bolisetty, Karel Allegaert, Anke Raaijmakers

**Affiliations:** 1 Department of Neonatology, Royal North Shore Hospital, St Leonards, NSW 2065, Australia; 2 University of Sydney Northern Clinical School, Royal North Shore Hospital, St Leonards, NSW 2065, Australia; 3 Department of Newborn Care, Royal Hospital for Women, Randwick, NSW 2031, Australia; srinivas.bolisetty@health.nsw.gov.au; 4 School of Women’s and Children’s Health, University of New South Wales, Kensington, NSW 2031, Australia; anke.raaijmakers@health.nsw.gov.au; 5 Department of Development and Regeneration, KU Leuven, 3000 Leuven, Belgium; karel.allegaert@kuleuven.be; 6 Department of Pharmaceutical and Pharmacological Sciences, KU Leuven, 3000 Leuven, Belgium; 7 Department of Hospital Pharmacy, Erasmus MC, 3015 GD Rotterdam, The Netherlands; 8 Department of Paediatric Nephrology, Sydney Children’s Hospital, Randwick, NSW 2031, Australia

**Keywords:** neonate, prematurity, acute kidney injury, chronic kidney disease, pharmacology

## Abstract

In neonates, estimation of the glomerular filtration rate is problematic, and assessment of renal impairment is challenging. Serum creatinine is a widely used marker, and urine output is an important vital parameter monitored in intensive care settings, particularly in unwell neonates. However, the rapid changes after birth with adaptation to the extrauterine environment is a unique situation in which absolute serum creatinine is not a reliable indicator of renal function. A rise in serum creatinine from the previous value during the neonatal period can be a result of worsening renal function in neonates but is dependent on many other factors. In addition, urine output can be difficult to measure in sick neonates during their intensive care stay. Despite a high prevalence of acute kidney injury (AKI) in preterm and/or unwell infants, the current definitions are not straightforward and do not take postnatal adaptation processes into account. The management of AKI is challenging in very young and small patients because the assessment of fluid status as well as balancing nutritional needs with fluid restriction can be problematic. The Australian Neonatal Medicines Formulary provides advice on drug dosing in the face of reduced renal function in neonates. Predictors (or long-term outcome, or recovery) after AKI diagnosis are still poorly described. Therefore, the diagnosis of neonatal AKI needs to be documented and transferred to the paediatrician responsible for the follow-up of the child. This educational review aims to give a perspective on neonatal kidney function and AKI, the relation of fluid balance and creatinine, the management of neonatal AKI and the consequences for drug dosing and long-term outcomes.

## 1. Introduction

**Figure 1:**
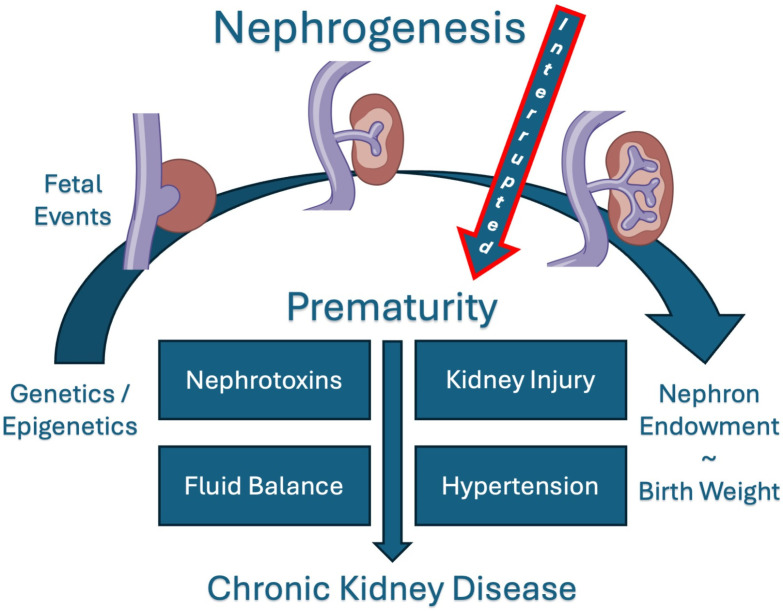
Nephrogenesis interruption by prematurity and its relation to chronic kidney disease. Nephron endowment is affected by birth weight and prematurity. Birthweight and nephron endowment have a direct positive correlation. The effect of prematurity on chronic kidney disease is multifactorial.

Nephrogenesis in utero continues till ~36 weeks of gestation, and no new nephrons are formed thereafter. Preterm birth partly hinders this process [1]. Preterm infants born prior to 36 weeks of gestation are born with reduced nephron numbers and potential suboptimal renal performance throughout life [2,3]. According to the Developmental Origins of Health and Disease (DOHaD) concept, renal insults in utero or early life may lead to reduced nephron mass, resulting in compromised organ development with altered function and morphology and, eventually, hypertension, proteinuria and possibly later chronic kidney disease (CKD) (Figure 1) [2,3]. Moreover, premature infants are commonly exposed to additional stressors (nephrotoxic drugs, sepsis, suboptimal nutrition leading to extrauterine growth failure, hypo/hyperoxygenation, etc.), further reducing nephron mass with potential consequences for their (long-term) kidney function. Therefore, there is a need to safeguard kidney development and function after preterm birth.

Neonatal acute kidney injury consists of a decline in GFR marked by a rise in creatinine compared to a previous ‘steady state’ [4]. Although awareness around neonatal kidney injury and protection is rising [5,6], neonatal serum creatinine in the first days of life is not yet a reliable indicator of renal function and reflects maternal values initially, then fluid changes and hydration status [7,8]. Despite a high prevalence of acute kidney injury in these infants, current definitions are not conducive to obvious recognition and do not take fluid balance into account. This educational review aims to give a perspective on neonatal kidney function and injury, the relation with fluid balance, the management of AKI and the consequences for drug dosing and long-term outcomes.

## 2. Postnatal Kidney Development and Function

Nephrogenesis is partly hindered and affected by preterm birth (Figure 1). Although recent evidence suggests ongoing, albeit reduced, nephrogenesis after birth in premature infants [9,10], the quantity and quality of these newly formed nephrons need further investigation [11]. Renal blood flow and GFR are low after birth (i.e., GFR ~10–30 mL/min/1.73 m^2^ at term, even lower with decreasing gestational age (GA)), then increase rapidly every day with decreasing renal vascular resistance and increased cardiac output [12]. Because the kidneys mature by vasoreactivity and as a function of the transitional circulation in the neonatal period [13], postnatal changes in renal blood flow and glomerular filtration are impaired in the event of, e.g., ventilation, sepsis or asphyxia and altered by fluid management and methylxanthines [14]. Patterns of GFR maturation in term and preterm neonates have been described using different data sources and modelling, which has resulted in reference values that consider postnatal maturation at different GAs [12,15]. Because GFR measurements via the clearance of exogenous markers are non-practical, creatinine-based GFR estimations using the Schwartz formula (eGFR [mL/min/1.73 m^2^] = (k × length [cm]/serum creatinine [mg/dL]) optimised for neonates utilise adjusted k-coefficients of 0.31 or 0.33 (instead of 0.45 as used in adults) to better represent body habitus and muscle mass [12,16].

After the initial rapid changes, GFR then slowly reaches full maturity over about two years, when the glomerular capillary tufts and tubules lengthen with increasing functional demand [17]. Early events, prematurity itself and co-morbidities, coupled with the underlying prematurity-related nephron deficit, may result in lifelong reduced functional kidney capacity. According to the original Brenner hypothesis, the compensatory hyperfiltration further damages individual nephrons, leading to the progressive loss of more nephrons and, consequently, to proteinuria, (further) reduced renal function, high blood pressure and, eventually, chronic kidney disease (Figure 1, [18]).

## 3. Fluid Balance and Neonatal Kidney Function

The placenta primarily handles creatinine in the foetus and is transported across the placenta in a bidirectional fashion by simple passive diffusion. Therefore, foetal and neonatal concentrations of creatinine reflect maternal serum creatinine immediately after birth in both preterm and term neonates [19,20]. However, a rise in serum creatinine from the initial value during the neonatal period can be the result of normal physiology or worsening renal function in the neonate but may also reflect many other factors like fluid balance [7]. Physiological postnatal volume contraction, mainly from transepidermal water loss, as evidenced by postnatal weight loss and hypernatraemia, is postulated to result in the higher concentration of creatinine in the remaining extra- and intravascular space [8]. While GFR and endogenous creatinine production are still very low, serum creatinine concentration consequently remains static initially, or, in the youngest infants with the largest extracellular fluid compartment, rises proportionally to weight loss [21]. In the subsequent weeks, vascular resistance falls and renal blood flow and GFR increase, resulting in a slow, steady approach of serum creatinine concentration to the normal value of young children around 2 years of age [22]. Reference values for serum creatinine in the categories of the gestational and postnatal ages represent the dynamic changes in the immediate postnatal period [21]. Laboratory reference values for serum creatinine may also vary depending on the assay; measurements using the Jaffé method are expected to be higher than those using enzymatic methods [23].

The understanding of physiological changes in total body water and fluid shifts after birth is a key component of managing sick newborns in the setting of acute neonatal intensive care. However, assessing fluid balance, particularly in a preterm infant, is challenging and dependent on many factors including (gestational and postnatal) age, insensible losses and incubator-mediated humidity [24] and is not approached uniformly by all clinicians [25]. Fluid overload, often evident as the absence of physiological weight loss in the first days after birth, should be considered a pathological state that needs to be prevented/treated [24,26,27], as evidenced by the large retrospective cohort study where for every 1% of positive fluid balance, the odds of adverse outcomes increased by 12% or more [28]. Judicious management of fluid intake to achieve optimal fluid balance is key as excessive dehydration may also cause potential harm, as recently illustrated in neonates receiving therapeutic hypothermia after moderate-to-severe asphyxia [29] and by the association of intraventricular haemorrhage with excessive weight loss in extremely preterm neonates [27]. Therefore, the relationship between fluid balance and outcome is U-shaped, with both excessive and lack of sufficient weight loss associated with increased risk of morbidity and mortality. As there is a lack of prospectively obtained evidence for normal values of fluid balance in the first two weeks of life, clinicians pragmatically use weight loss as a proxy to guide fluid management in this time of rapid fluid shifts. Nomograms for weight loss for healthy breastfed neonates demonstrate a weight nadir between 7 and 12% at around 72 h of life [30]. In extreme preterm infants, 9–15% weight loss in the first days of life appears to be optimal for the prevention of death and later neurodevelopmental impairment [31].

## 4. Definition of AKI in Neonates

Both acute kidney injury (AKI) and subsequent chronic kidney disease (CKD) occur frequently in the NICU population [6] but are often underrecognised or underestimated in comparison to other organ dysfunction such as that of the lungs, heart, retina or brain. There is a window of opportunity to preserve and improve kidney function in preterm neonates by instituting kidney protective measures during their NICU stay, firstly based on mitigation strategies [5,32] then careful management of the premature infant when complications occur.

Renal function is assessed as the glomerular filtration rate (GFR), traditionally estimated from serum creatinine values. Despite its limitations, serum creatinine is a widely available marker. Variation in urine output is an important parameter for the diagnosis of AKI and renal function and is routinely monitored in neonatal intensive care unit (NICU) settings, particularly in unwell or preterm neonates [4]. Accurately estimating urine output in the smallest neonates can be challenging due to the erratic voiding pattern, non-availability of appropriately sized in-dwelling urinary catheters and imprecision of weighing of diapers or absorbent material placed in diapers.

The Assessment of Worldwide Acute Kidney injury Epidemiology in Neonates (AWAKEN) study is the largest neonatal AKI study to date [6]. It is the first multicentre, multinational, retrospective cohort study of critically ill neonates of all gestational age groups in four countries (Australia, Canada, India, USA). The study used modified Kidney Disease: Improving Global Outcomes (KDIGO) criteria for the definition of AKI (Table 1) and showed that about 30% of sick neonates admitted to the NICU develop AKI. The incidence of AKI varied by gestational age group, occurring in 48% of neonates born at <29 weeks, 18% born at 29 weeks to <36 weeks, and 37% of neonates born at ≥36 weeks. Neonates with AKI had an independently higher mortality than those without AKI (10% vs 1%) and a longer length of hospital stay [6]. Upon recommendations by the AWAKEN study group and KDIGO workgroup, many neonatal clinicians and researchers have adopted the use of the definition of AKI per these modified KDIGO criteria, although they remain empirical to date and have yet to be evaluated in a large neonatal study (Table 1) [4,7].

Fluid balance will have an impact on the diagnosis of AKI, as volume contraction and fluid redistribution after birth result in the frequently seen upward slope of serum creatinine concentration. Similarly, fluid overload will affect the serum concentration of creatinine by showing a static trend or lesser increase even in the presence of AKI. Using the formula [serum creatinine × (% change of birthweight divided by total body water)] takes the redistribution of creatinine with changing fluid balance into account and corrects serum creatinine concentration [8]. Applying the fluid correction of serum creatinine has been shown to increase the number of (preterm) infants with an AKI diagnosis [34].

Creatinine is a relatively late marker of AKI in neonates and is mainly reflective of (acutely or chronically) impaired function rather than just acute injury [35]; however, it remains the most widely used. Other biomarkers such as neutrophil gelatinase-associated lipocalin (NGAL) indicate injury to the tubular apparatus before creatinine starts to rise. Importantly, the use of urine NGAL can be a non-invasive method of detecting the onset of renal impairment [36,37]. It could potentially also aid management decisions, e.g., differentiating between fluid needs or renal replacement therapy during AKI [38], although this concept remains to be tested in neonates. Cystatin C is another marker for renal function which has recently gained some traction in the diagnosis of neonatal AKI, as it is independent of patient size or muscle mass and does not cross the placenta [39]. In comparison with measured GFR, cystatin C appears to overestimate glomerular filtration in neonates and warrants further validation [40]. Newer, non-invasive technology such as near-infrared spectroscopy (NIRS) shows some promise by flagging decreased local tissue oxygenation as an early sign of AKI [41]. There is a need to establish alternative (urine) biomarkers of AKI that, ideally, indicate evolving kidney injury early and specifically.

## 5. Prevention and Management of Neonatal AKI

With the advancement in neonatal medicine and the improvement in the survival of extremely premature infants and critically unwell neonates, more instances of AKI have been diagnosed and potentially deleterious long- and short-term effects recognised [42,43]. Despite these advances, there remains room for improvement in the timely recognition and documentation of neonatal AKI, particularly in patients at the highest risk: extremely-low-birth-weight infants and term neonates exposed to birth asphyxia. In view of the potentially deleterious short- and long-term effects of neonatal AKI, increasing the awareness of a multidisciplinary approach to prevention and management, including the early consultation of paediatric nephrology, is paramount [44,45,46].

### 5.1. How to Prevent Neonatal AKI

The first step in neonatal AKI management is prevention. In the style of antimicrobial stewardship programs, close surveillance of the use of nephrotoxic drugs and the monitoring of renal function have been shown to decrease the incidence of neonatal AKI. The baby NINJA (Nephrotoxic Injury Negated Just-In-Time Action) quality improvement initiative introduced daily screening reports for infants receiving nephrotoxic medication and regular measurements of serum creatinine, with the effect of decreasing exposure to high-risk nephrotoxic medication and instances of AKI [5]. This type of pharmacovigilance has since been replicated by others with good success [47]. Specific decisions around the clinical management of preterm and sick newborn infants, such as strategies to avoid or shorten courses of invasive ventilation [48] and judicious fluid management, can mitigate the risk of AKI [49,50]. The presence of a patent ductus arteriosus (PDA) is associated with an increased risk of AKI [51], particularly when the haemodynamic effects of the PDA compromise respiratory status [52]. While PDA treatment with non-steroidal anti-inflammatory drugs (NSAIDs) can cause AKI, the treatment of PDA using these drugs may ultimately decrease instances of moderate-to-severe AKI [52]. Methylxanthines, which are used in the NICU to treat apnoea of prematurity, are the only pharmacotherapy known to reduce the risk of AKI in different neonatal populations. The early use of caffeine is associated with a lower rate of AKI in premature infants [53,54]. A single dose of aminophylline has been shown to significantly reduce the incidence of AKI in term neonates with birth asphyxia [55], although the benefit is less clear in the context of therapeutic hypothermia [56]. The surge in the concepts of Artificial Intelligence and machine learning shows promise in better prediction and detection for neonatal AKI; however, these novel concepts have yet to prove that they improve the short- or long-term outcomes of premature or sick term infants [57,58].

### 5.2. How to Manage Neonatal AKI

The management of AKI in small neonates is challenging due to diagnostic and technical reasons and requires meticulous attention to detail [59,60]. The principle of the conservative management of neonatal AKI aims to address underlying causes, maintain appropriate fluid intake for glycaemic control and nutrition while avoiding fluid overload and adjust electrolytes to maintain a biochemically safe state while waiting for renal function to recover from the acute insult. Close observations of vital parameters including blood pressure and the monitoring of input and output are paramount in the acute phase of AKI. Fluid balance can be challenging to estimate accurately. Fluid intake (intravenous fluids, enteral nutrition, blood products, medication and flushes) is easier to quantify than output, as urine output is often measured only by weighing diapers, stool is equally hard to quantify and insensible losses are very dynamic. In the absence of accurate measurements of input and output, once or twice daily weights as a proxy should be the minimum standard and has been used as a feasible and reliable surrogate for fluid balance. Fluid balance is calculated based on the comparison of daily weight with birth weight (or last known stable weight before onset of AKI): % change = (current weight − birth weight)/birth weight × 100). In the first two weeks of life, weight loss outside of the optimal range quoted for extremely preterm neonates or breastfed term infants (see Section 3) is to be considered fluid overload or excessive dehydration. After week 2, 20 g/kg/day is considered an appropriate and optimal weight gain until term-corrected age and can be used as a proxy to assess and manage fluid balance [61]. Arterial access allows close monitoring of electrolytes and renal function (with the added benefit of invasive blood-pressure monitoring) while avoiding repeat skin breaks in fragile premature infants [62].

Obvious causes for decreased renal function can be assessed using ultrasound of the kidneys and urinary tract and should be addressed as a priority; filling the intravascular space (isotonic fluid or blood products,) and treatment of hypotension in pre-renal cases of AKI will improve renal perfusion. Concomitant measurement of serum and serum sodium for the calculation of fractional excretion of sodium (FENa) can further assist in differentiating between pre-renal and renal AKI. There is no evidence to support low “renal” doses of dopamine infusions to prevent or improve AKI in premature or sick neonates [63]; rather, inotropic and vasopressor support should be chosen according to the underlying cause of low blood pressure and/or poor cardiac output [64]. Signs of renal vein thrombosis (typically a triad of haematuria, flank mass and thrombocytopenia) [65] or post-renal obstructions should be investigated and treated accordingly. If urine output is preserved, i.e., renal tubules are still responsive, it is worthwhile to attempt to increase urine production with diuretics after a fluid challenge. Relatively large doses of furosemide or stronger diuretics such as metolazone may be required to deliver effective concentrations to the peritubular environment. During acute tubular necrosis with anuria, repeat or high doses of furosemide are unlikely to yield urine production until tubular recovery, and harmful side effects such as ototoxicity will prevail [66].

After the initial correction of intravascular fluid status, ongoing fluid therapy is titrated to maintain hydration, euglycaemia and replacement of losses until renal function starts normalising. Fluid overload should be avoided at all costs, as the evidence of detrimental effects thereof becomes more obvious [67]. In the absence of any urine production, fluid intake is consequently limited to insensible water loss, the majority of which will be via transepidermal water loss (TWL). The estimation of TWL is a function of gestational and chronological age, ambient humidity and temperature and skin integrity. Practical approaches to estimate TWL include the use of published tables or apps [49,68,69]. Whenever possible, the maintenance of hydration should consist of, or at least include some, enteral nutrition.

Mild-to-moderate hyponatremia is generally dilutional rather than reflective of actual loss of sodium and is safe to be treated with fluid restriction alone. Higher potassium levels are more commonly seen in preterm neonates than in other paediatric populations [70]. Nevertheless, hyperkalaemia in the face of renal dysfunction is an indication for urgent intervention before signs of cardiac toxicity emerge. Only loop diuretics and exchange resins remove potassium from the body, while all other ways of treatment of hyperkalaemia either shift potassium into cells (salbutamol, sodium bicarbonate, glucose insulin infusion) or stabilise the myocardium (intravenous calcium). Hyperphosphatemia is primarily addressed with dietary restriction, for which mother’s own milk, if available, is preferable over renal formula milk, as the phosphate content in human milk is naturally low [71], and because the cow’s milk base of renal formulae such as Renastart poses a relevant risk of necrotising enterocolitis in the premature infant. Equally, the safety of oral phosphate binders other than water-soluble calcium compounds has not been established for the immature gastrointestinal system of preterm neonates. Hypocalcaemia should be corrected before sodium bicarbonate is given to treat metabolic acidosis, because the correction of acidosis will further lower the levels of ionised calcium.

Once urine production starts to re-establish, replacement should be 1:1 if the patient is euvolemic, or proportionally less in the case of fluid overload, to achieve a negative balance. The type of fluid administered should match the losses and sodium content of the patient. Once creatinine starts to decline and clinical condition improves, tight management of input and output can be relaxed in favour of increasing enteral nutrition.

Most instances of neonatal AKI have a favourable prognosis, and conservative management aims to restore and maintain homeostasis until renal function recovers. However, certain circumstances may require escalation to renal replacement therapies (RRTs): life-threatening hyperkalaemia, or other severe intractable electrolyte disbalances or acidosis; severe fluid overload; severe uraemia or inability to reinstitute nutrition due to prolonged oligo- or anuria. Until relatively recently, renal replacement therapy options for small newborns and premature infants were limited to peritoneal dialysis, although even this option is challenging in the smallest infants. Technical advances have led to the development of machines for extracorporeal kidney support suitable for small patient size with minimisation of volumes in extracorporeal circuits, adjustment to smaller vascular-access catheters and careful considerations of anticoagulation [72]. The management of neonatal AKI requires close collaboration between neonatologist and paediatric nephrologist with neonatal expertise and must include discussions of RRT options if conservative management cannot see the patient through to recovery.

### 5.3. How to Adapt Drug Dosing in Neonatal AKI

Many drugs used in neonates are renally cleared, and the accumulation of the substances and their active or toxic metabolites can affect a variety of organ (kidney and other) and metabolic functions [73]. Studies have highlighted the potential toxic effects of various drugs that premature infants are exposed to both in utero and postnatally, namely, antimicrobials of the class of aminoglycosides and glycopeptides, cephalosporins, antifungals, virostatic drugs such as acyclovir and ganciclovir, non-steroidal inflammatory drugs and ACE inhibitors [32,73]. Nephrotoxic side effects can further decrease renal function and hamper the already-reduced potential of ongoing nephrogenesis after premature birth [74]. Even in the paediatric setting, dosing adjustments for renally cleared drugs are commonly derived from adult recommendations rather than paediatric clinical studies [75,76]. In the unique context of the newborn kidney, where GFR is physiologically low immediately after birth then slowly increases filtration and tubular function over the first few weeks and months, there is a dearth of evidence for well-informed guidance in the adjustment of dosing with decreased renal function. There are reviews available with general considerations of dosing and therapeutic monitoring [77,78], but none really addresses the question of dosing alterations when renal function is impaired in a pragmatic overview [13]. Machine learning and model-based approaches (population pharmacokinetics or physiological pharmacokinetics) may present opportunities to optimise neonatal drug dosing without the extensive exercise of studying compounds individually [7,79,80].

The Australian Neonatal Medicines Formulary (ANMF) is a free resource providing evidence-based, consensus-driven monographs for many medicines commonly used in neonates [81]. The documents are produced by an expert group of neonatologists and pharmacists after thorough review of the literature relevant to the use of drugs in the neonate. The ANMF group consensus is to use the modified KDIGO criteria for the definition of AKI and the diagnosis of AKI as a signal to adapt drug dosing in the clinical setting.

Every monograph of the ANMF contains a section with advice on dosing in infants with impaired renal function. For example, the monograph for vancomycin administration as continuous infusion advises to give an initial loading dose, then dosing of the infusion according to serum creatinine concentration and corrected gestational age. Different strengths of the standard infusion concentrations can be chosen depending on the fluid requirements, including an option for fluid-restricted patients. The monograph then also gives guidance on the frequency of therapeutic drug-level monitoring and dose adjustment based on the available evidence on pharmacokinetics in neonates [82]. However, only 16% of the over 200 ANMF monographs contain clear instructions on how to adjust dosing in renal impairment, mostly for drugs with well-known renal toxicity such as aminoglycosides. In this space of missing evidence, the first and most important step for the clinician is the recognition of reduced renal function, which is why ANMF has developed a document to facilitate a pragmatic approach to assessing the degree of AKI in neonates. Secondly and practically, pre-emptive review of current medications helps minimise the concurrent use and length of exposure to nephrotoxic medications [5,32].

### 5.4. Follow-Up of Neonates Post-AKI

Over the past decade, evidence from population-based and larger cohort studies has increasingly made it clear that low birth weight with or without associated prematurity and prematurity-related morbidities increase the risk of impaired kidney function and arterial hypertension from young adulthood [2,3]. In a meta-analysis, preterm-born survivors had decreased glomerular filtration, increased albuminuria, decreased kidney size and volume and higher blood pressure, even though laboratory markers such as serum creatinine and cystatin C were not (yet) worse than in full-term controls [83]. Young adults born preterm have a 70% increased relative risk of chronic kidney disease, and birth before 28 weeks approximately doubles the risk of high blood pressure compared to those born at term [3,84]. Episodes of neonatal AKI negatively impact blood pressure and renal function later in life and are frequently compounded by the adverse long-term effects of prematurity on renal health [85,86,87].

While the follow-up of neurodevelopmental sequelae has a firm place in the care of the NICU graduate, awareness of the need for the follow-up of kidney health is still lacking [1,3,84]. The first step towards a more structured follow-up of long-term renal sequelae is the recognition and documentation of the AKI diagnosis in the neonatal period and the transfer of the information into the discharge summary to the paediatrician or family physician responsible for the follow-up of the child [88]. There are several initiatives, including automatic alert systems, to improve this surveillance [89,90]. Many medical record systems do not include the history of prematurity and associated morbidities in medical records beyond infancy. In the Australian state of New South Wales, the electronic medical health record, which every resident can opt into for improved transparency across health care providers and areas, does not include information on gestational age at birth or birth weight. This is in addition to the lack of awareness of the developmental aspects of how birth weight and nephron numbers result in late renal sequalae for prematurely born individuals [91], particularly in the space of general practice and family medicine. There is an important need for initiatives to improve education on, and recommendations for, the follow-up of renal health for former premature infants or infants with a history of neonatal kidney problems. The current Australian guidelines for the follow-up of children born very preterm acknowledge the increased cardiovascular risk of elevated blood pressure in this population [92]. A recent consensus statement from the Neonatal Kidney Health Workshop issued consensus recommendations that acknowledge the barriers to kidney health assessment throughout childhood and identify important gaps and research priorities in this area [93]. The Neonatal Kidney Collaborative, with its mission that it “improves the health of newborns with or at risk of kidney disease through multidisciplinary collaborative research, advocacy and education” [94], has been at the forefront of advocacy for the inclusion of (short- and long-term) renal outcomes in all neonatal clinical trials. This harbours the potential to bring screening for long-term kidney health to the same standard as pulmonary and neurodevelopmental outcomes [95].

## 6. Conclusions

Definition of renal impairment in neonates is challenging and needs more awareness, both during the NICU stay as well as with respect to the clinicians providing long-term follow-up for these infants. In neonates, the estimation of GFR is problematic, and care needs to be taken to include fluid balance in AKI considerations. Serum creatinine is a widely available marker, and urine output is an important vital parameter monitored in intensive care settings, particularly in an unwell neonate. However, a rise in serum creatinine from the previous value requires the context of total fluid balance for a meaningful assessment of kidney function. Despite a high prevalence of acute kidney injury in these infants, more research is needed to develop clinical parameters and tools to make renal dose adjustments and bedside fluid assessments more feasible. The diagnosis of AKI in the neonatal period needs to be documented and transferred to the paediatrician responsible for the follow-up of the child.

## Figures and Tables

**Table 1:** Neonatal Acute Injury definitions. Adapted from the Kidney Disease: Improving Global Outcomes (KDIGO) workgroup [4]. AKI is defined by creatinine and/or urine output criteria.

AKI Stage	Serum Creatinine (μmol/L)	Urine Output
0	No change in serum creatinine or rise < 26.5 μmol/L (<0.3 mg/dL) ^1^	>1 mL/kg/h
1	SCr rise ≥ 26.5 μmol/L (>0.3 mg/dL) within 48 h or SCr rise ≥ 1.5–1.9× previous SCr value within 7 days ^1^	>0.5 and ≤1 mL/kg/h
2	SCr rise ≥ 2 to 2.9× previous SCr ^1^	>0.3 and ≤0.5 mL/kg/h
3	SCr rise ≥ 3× previous SCr or renal replacement therapy ^1^	≤0.3 mL/kg/h

^1^ Consider fluid correction of serum creatinine: SCr × [total body water + (current weight − birth weight)]/total body weight)] [8], where total body water per gestational age is estimated as follows: 0.9 × weight for <26 weeks; 0.85 × weight for 26–32 weeks; 0.8 × weight for 32–36 weeks; 0.75 × weight for >36 weeks [33].
